# The possible impact of persistent virus infection on the function of the RNAi machinery in insects: a hypothesis

**DOI:** 10.3389/fphys.2013.00319

**Published:** 2013-11-05

**Authors:** Luc Swevers, Jozef Vanden Broeck, Guy Smagghe

**Affiliations:** ^1^Insect Molecular Genetics and Biotechnology, Institute of Biosciences and Applications, National Center for Scientific Research “Demokritos,”Athens, Greece; ^2^Molecular Developmental Physiology and Signal Transduction, Department of Biology, KU LeuvenLeuven, Belgium; ^3^Department of Crop Protection, Faculty of Bioscience Engineering, Ghent UniversityGhent, Belgium

**Keywords:** RNAi, persistent virus infection, RNA virus, insects, pest control, innate immune response

## Abstract

RNAi experiments in insects are characterized by great variability in efficiency; for instance beetles and locusts are very amenable to dsRNA-mediated gene silencing, while other insect groups, most notably lepidopterans, are more refractory to RNAi. Several factors can be forwarded that could affect the efficiency of RNAi, such as the composition and function of the intracellular RNAi machinery, the mechanism of dsRNA uptake, the presence of dsRNA- and siRNA-degrading enzymes and non-specific activation of the innate immune response. In this essay, we investigate the evidence whether persistent infection with RNA viruses could be a major factor that affects the response to exogenous dsRNA in insects. The occurrence of RNA viruses in different insect groups will be discussed, as well as several mechanisms by which viruses could interfere with the process of RNAi. Finally, the impact of RNA virus infection on the design of dsRNA-based insect control strategies will be considered.

## Introduction

The discovery that dsRNA can trigger silencing of homologous RNA sequences has revolutionized the analysis of gene function in a wide range of eukaryotic organisms such as plants, fungi, and metazoans (Carthew and Sontheimer, 2009; Jinek and Doudna, 2009; Moazed, 2009; Siomi and Siomi, 2009a). This technique, called RNA interference (RNAi), has also been applied to insects, to study gene function in basic research, and in agriculture as a strategy to control insect pests (Huvenne and Smagghe, 2010). In most of these studies, gene silencing is achieved by injection of dsRNA in the hemolymph of the insect, from where it is taken up by the cells to exert its silencing effects. When those studies expanded, however, it became clear that some insects showed a very specific and potent response to injected dsRNA while much less clear results were obtained in other cases (Terenius et al., 2011). This has led to an inquiry into the processes that are necessary to achieve successful RNAi *in vivo* (Terenius et al., 2011).

## Types of RNAi: intracellular, systemic, and environmental

As a starting point to explain the differential success of RNAi in different insects, one has to consider the method of delivery of dsRNA. In the case of intracellular RNAi, dsRNA is produced or introduced efficiently inside the cells; examples are injection of dsRNA into preblastoderm embryos (before formation of cellular membranes; Quan et al., 2002; Yamaguchi et al., 2011), production of hairpin RNAs (folding into dsRNA structures) in transgenic animals (Dai et al., 2007; Kanginakudru et al., 2007) and intracellular production of RNA viruses which form replication intermediates with dsRNA structures (Flynt et al., 2009; Siu et al., 2011). Another example is transfection of tissue culture cells with dsRNA-cationic lipid complexes because this procedure allows efficient uptake as is the case for DNA transfection (Kolliopoulou and Swevers, 2013). In the majority of cases of intracellular production of dsRNA or introduction of dsRNA in tissue culture cells, high efficiency of gene silencing is achieved, indicating that the intracellular RNAi machinery can work efficiently in most eukaryotic cells.

Expression studies have revealed ubiquitous expression of the mRNAs that encode the intracellular components of the RNAi machinery. In the silkmoth, *Bombyx mori*, the basic machinery of the three small RNA pathways [microRNA (miRNA), small interfering RNA (siRNA) and PIWI-associated RNA (piRNA)] is expressed in all tissues investigated such as epidermis, fat body, gut, muscle, Malpighian tubules, silk gland, gonads, and hemocytes (Swevers et al., 2011). In *Drosophila*, genetic studies established separate functions of the different small RNA pathways that are executed by separate, although partially overlapping, protein complexes (Carthew and Sontheimer, 2009; Jinek and Doudna, 2009; Moazed, 2009; Siomi and Siomi, 2009a). Thus, miRNAs originate from endogenous genes and are involved in the regulation of development and physiological processes while siRNAs are generated from exogenous and endogenous long dsRNAs (dsRNA structures from parasitic nucleic acids such as infecting viruses and transposable elements) and harnessed to combat viral and transposon RNAs. Both pathways are characterized by different Dicer enzymes, Argonaute proteins and dsRNA-binding proteins (Drosha/Dcr-1, Ago-1, and Pasha/Loqs for miRNAs; Dcr-2, Ago-2, and R2D2 for siRNAs), although cross-talk between the two pathways is possible (Tomari et al., 2007). A third, Dicer-independent, pathway, characterized by the production of piRNAs, was originally thought to be expressed only in the germline to serve as protection against transposons and mobile elements (Vagin et al., 2006), but later studies firmly established its expression in somatic tissues as well, not only in *Drosophila* (Yan et al., 2011) but also in the silkmoth (Kolliopoulou and Swevers, 2013), and implicated its involvement in antiviral defense in mosquitoes (Hess et al., 2011; Morazzani et al., 2012; Vodovar et al., 2012).

While in worms, fungi and plants RNA-dependent RNA polymerases (RdRps) are part of the RNAi machinery, this is not the case for insects (or mammals) (Gordon and Waterhouse, 2007). In *C. elegans*, RdRps can function to generate secondary siRNAs that amplify the silencing signal (Miska and Ahringer, 2007). Because of the absence of RdRP genes, it can be speculated that RNAi efficiency in insects may be less robust than in *C. elegans*. However, other organisms with RdRp genes, such as plant nematodes or mites, do not respond efficiently to injection of dsRNA (Khila and Grbić, 2007; Dalzell et al., 2011), indicating that the presence of this gene is not sufficient for efficient systemic RNAi.

While the RNAi machinery seems to be expressed and functional in most tissues of insects, most reverse genetic studies using RNAi in insects are based on the injection of dsRNA in the body cavity (hemolymph) of the insects (Terenius et al., 2011). In this review, this process, based on the efficient uptake of dsRNA from the hemolymph to cause intracellular silencing, is called “systemic RNAi.” This definition does not take into account whether cells, after uptake of dsRNA, can further spread the initial silencing trigger or its effectors throughout the whole organism, which is incorporated in other definitions of systemic RNAi, such as that used by Zhuang and Hunter (2012). The definition used in the review is based on the practical question: “does injection of dsRNA in the hemolymph cause silencing?” and avoids the question whether dsRNA-based silencing occurs only in cells that have directly taken up dsRNA during the initial injection or whether it can subsequently spread to cells that have not directly taken up the injected dsRNA but received it via an intercellular transfer mechanism.

Another method is the administration of dsRNA through feeding (“environmental RNAi”); this administration route is particularly relevant with respect to the development of RNAi-based methods for pest control (Price and Gatehouse, 2008). Again in this review, the term “environmental RNAi” is based on the practical question: “does feeding of dsRNA cause silencing?” without going into mechanistical details (Whangbo and Hunter, 2008).

In the worm *C. elegans*, the gene *sid-1* has been identified that is responsible for the systemic effects of RNAi; it encodes a transporter protein that is responsible for the transport of dsRNA across the plasmamembrane to the cytoplasm of the cells (Hunter et al., 2006). While *sid-1* homologs have been identified in insects (with the notable exception of Diptera; Gordon and Waterhouse, 2007), it is not clear whether these are involved in the transport of dsRNA or in other transport functions (Tomoyasu et al., 2008). In *Drosophila*-derived S2 cells, scavenger receptors and the endocytosis machinery have been found to be implicated in the uptake of dsRNA and the trigger of extracellular RNAi (Saleh et al., 2006; Ulvila et al., 2006). In another study, it was shown that extracellular dsRNA can trigger RNAi effects in S2 cells through uptake by endocytosis and the association of the late endosomes with RNA-processing GW bodies (Lee et al., 2009), which are enriched for the RNAi machinery (Schneider et al., 2006). However, these are experiments with tissue culture cells and the mechanism by which dsRNA is taken up by the cells *in vivo* remains unknown. In experiments with the coleopteran *Diabrotica virgifera*, in which dsRNA was administered through feeding, it was demonstrated that the silencing trigger can spread efficiently from the gut epithelium to other tissues, by unknown mechanism (Bolognesi et al., 2012).

## Variability of systemic RNAi in insects

There exists quite a variability regarding the robustness of silencing effects triggered by injection or feeding of dsRNA in insects of different orders. In coleopteran (beetle) species, systemic RNAi can be applied very efficiently. This robustness actually resulted in the rise of the red flour beetle, *Tribolium castaneum*, as a model for the study of physiological and developmental processes in insects (Konopova and Jindra, 2007; Tan and Palli, 2008). Regarding coleopteran pests, it has been demonstrated that feeding of dsRNA targeting essential genes results in toxic effects and mortality in the corn rootworm, *Diabrotica virgifera*, and the Colorado potato beetle, *Leptinotarsa decemlineata* (Baum et al., 2007; Zhu et al., 2011). In the case of the corn rootworm, median lethal concentrations of the most active dsRNAs (targeting proteins involved in vesicular transport) of less than 1 ng/cm^2^ were obtained (Baum et al., 2007).

In cockroaches (Dictyoptera) and locusts (Orthoptera) injection of dsRNA can also trigger potent responses. In the cockroach, systemic RNAi has been used to unravel the ecdysone regulatory cascade that governs moulting and metamorphosis and the regulation of oogenesis (Ciudad et al., 2007; Mané-Padrós et al., 2010). In locusts, injection of dsRNA has been applied to elucidate the role of multiple genes implicated in neuronal and/or endocrine signaling pathways by RNAi, identifying their activities in the control of important biological processes, such as lipophilic hormone biosynthesis (Marchal et al., 2011a,b, 2012; Van Wielendaele et al., 2012, 2013c), food uptake (Van Wielendaele et al., 2012, 2013a; Dillen et al., 2013), digestion (van Hoef et al., 2011), reproduction (Badisco et al., 2011; Van Wielendaele et al., 2013b,c), and gregarisation behavior (Ott et al., 2012). Knockdown of this wide variety of transcripts revealed that many locust tissues are affected by the RNAi response, which eventually can result in significant phenotypic effects. In locusts, it was established that doses in the range of a few ng per gram tissue can already result in silencing (Wynant et al., 2012). A reduced response of RNAi was, however, observed in the adult male and female reproductive systems, which could be correlated to lower levels of expression of *Dicer-2* and *Ago-2* (Wynant et al., 2012). In addition, the protein turnover rate was also identified as an important parameter to achieve a phenotypic effect caused by RNAi (Ott et al., 2012).

In Diptera (flies and mosquitoes), injection of dsRNA has also been applied successfully to achieve gene silencing. In adult mosquitoes, systemic RNAi has been used to knock-down and investigate the functional role of genes, for example in the immune response against parasites and in the olfactory response (Biessmann et al., 2010; Pelletier et al., 2010; Bahia et al., 2011). It is noted, however, that much higher relative doses are used (200–800 ng dsRNA per mosquito in these studies) than in the case of the coleopterans and locusts discussed above. Furthermore, some tissues, such as salivary glands, seem rather refractory to systemic RNAi and silencing is only achieved after administration of high doses (Boissona et al., 2006). In *Drosophila*, successful RNAi has also been obtained by injection of dsRNA in adults (Dzitoyeva et al., 2001; Goto et al., 2003; Petruk et al., 2006). However, in larvae dsRNA-mediated gene silencing could not be achieved in most tissues, with the exception of the hemocytes (Miller et al., 2008).

In bees (Hymenoptera), injection of dsRNA has also resulted in gene silencing effects to analyze physiological and developmental processes such as sex determination, molting and the immune response (Gempe et al., 2009; Elias-Neto et al., 2010; You et al., 2010). Typically, 5–20 μg of dsRNA were injected per bee to achieve gene silencing and phenotypic effects. DsRNA can also be applied by feeding and environmental RNAi has been used as a strategy to control pathogen infection (Hunter et al., 2010).

In lepidopteran insects (moths and butterflies), a great variability in success rate exists in the literature with respect to systemic and environmental RNAi efficiency. In the silkmoth, several studies were reported in which successful gene silencing was achieved (Ohnishi et al., 2006; Tian et al., 2010; Deng et al., 2012). Environmental RNAi has also been used to induce toxic effects in the cotton bollworm, *Helicoverpa armigera* (Mao et al., 2007). On the other hand, other studies claimed that RNAi through injection did not work in *Bombyx* and that other strategies of delivery need to be developed (Uhlirova et al., 2003; Marcus, 2005). To evaluate better the efficiency of RNAi in Lepidoptera, a database was constructed of RNAi studies which included also negative results (http://insectacentral.org/RNAi). The analysis of the results deposited in the database led to the conclusion that RNAi is not an efficient process in Lepidoptera but failed to identify conditions that need to be met to achieve success in RNAi (Terenius et al., 2011).

The use of RNAi in Hemiptera has a lot of attention because it could provide an effective new strategy to combat important hemipteran pests (aphids, whiteflies, planthoppers, leafhoppers), which feed on plants through sap-sucking and for which no effective Bt toxins for control are known (Chougule and Bonning, 2012). As is the case for the Lepidoptera, however, great variability in success of systemic and environmental RNAi against Hemiptera is reported, including influential studies that have shown clear success (Mutti et al., 2008) while other studies documented more limited efficacy to obtain a phenotype (Ghanim et al., 2007; Jaubert-Possamai et al., 2007; Shakesby et al., 2009). The inconsistency of results of RNAi in Hemiptera makes this approach currently inadequate for use in management of these pests (Chougule and Bonning, 2012).

## Factors that determine RNAi success

The variability in RNAi success rate in Lepidoptera and Hemiptera, two insect orders with a large number of important insect pests, invited research to identify the factors that limit RNAi efficacy. Identification of those factors may provide for future application of this approach in insect pest management programs.

Several factors were considered that explain the difference in RNAi success among insects of different groups (Terenius et al., 2011) and these will be discussed briefly.

### Expression levels of RNAi machinery

The higher RNAi efficacy in coleopteran insects compared with Lepidoptera could be explained by higher levels of expression of the relevant RNAi machinery (triggered by exogenous dsRNA; siRNA pathway) in the tissues of the former. Unfortunately, very little is known with respect to differences in expression of the RNAi machinery among different insects. In *Bombyx mori*, it was found that all core RNAi genes of the three small RNA pathways were broadly expressed in most tissues, with the exception of *r2d2*, which encodes a dsRNA-binding protein that acts as a cofactor of Dicer-2 in the siRNA pathway (Swevers et al., 2011). In *Drosophila, r2d2* was shown to be essential for RNAi and the immune response against RNA virus (Liu et al., 2003; Wang et al., 2006; Zambon et al., 2006). Nevertheless, it was shown that a potent RNAi response is triggered in *Bombyx*-derived Bm5 cells in the absence of *r2d2* (Kolliopoulou and Swevers, 2013). Thus, differences can exist among different insect species (and cell types) regarding the gene repertoire that is necessary for potent RNAi.

As mentioned before, the relatively low expression levels of *Dicer-2* and *Ago-2* in (adult) gonads could explain the lower RNAi efficiency in these tissues in *Schistocerca gregaria* (Wynant et al., 2012). The mosquito (*Aedes albopictus*)-derived C6/36 has also been shown to have defective Dicer-2 expression which could explain the relatively robust growth of arthropod-borne viruses in this cell line (Scott et al., 2010; see also below for a discussion of RNAi as antiviral defense mechanism). Interestingly, C6/36 cells (and other mosquito-derived cell lines) produce viral-derived piRNAs following RNA virus infection, indicating their possible involvement in the control of viral growth (Vodovar et al., 2012). Thus, a certain redundancy may exist with respect to the pathways that trigger RNAi in cells.

### Uptake of dsRNA from the extracellular medium

In *C. elegans*, genetic analysis has resulted in the identification of a gene that encodes a putative transmembrane transporter protein, SID-1, that is responsible for systemic RNAi, while a second gene, *sid-2*, was also found to be necessary for the uptake of dsRNA from the food (environmental RNAi; Whangbo and Hunter, 2008). As discussed before, homologs of *sid-1* were identified in insects but it is doubtful that they play a similar role (Tomoyasu et al., 2008). *Sid-1-like* genes in insects are more closely related to another gene in *C. elegans* that encodes a transmembrane reporter (*tag-130*) and silencing of *sid-1-like* genes in *Tribolium* did not interfere with systemic RNAi (Tomoyasu et al., 2008).

RNAi screens in *Drosophila*-derived S2 cells that aimed at the identification of genes necessary for silencing after addition of dsRNA at the extracellular medium (“dsRNA soaking”) revealed the involvement of scavenger receptors and the endocytosis pathway (Saleh et al., 2006; Ulvila et al., 2006). A later study revealed that multivesicular bodies or late endosomes can associate with GW bodies to induce gene silencing (Lee et al., 2009). This points at a bifurcation of the endocytosis pathway at the late endosome stage: either fusion with lysosomes and degradation of dsRNA, or association with RISC-containing GW bodies and induction of RNAi. This model seems to be confirmed from the analysis of the *systemic RNAi defective* (*sid*) series of mutants in *C. elegans* (Rocheleau, 2012).

Multivesicular bodies can also fuse with the plasma membrane (exocytosis); such exosomes can contain mRNAs and small RNAs and be transferred to recipient cells (Siomi and Siomi, 2009b). In mammals, it is speculated that natural transport systems exist that transport small RNAs as a means of intracellular communication (Wang et al., 2010; Arroyo et al., 2011; Vickers et al., 2011). It remains to be investigated whether such communication system also occurs in insects.

Regarding a natural role for dsRNA in the hemolymph, it is speculated that when cells are damaged or underwent apoptosis after viral infection, viral dsRNA can accumulate in the hemolymph and trigger a systemic RNAi response (Saleh et al., 2009; Merkling and van Rij, 2013). Genes that are required for silencing through extracellular dsRNA soaking in S2 cells were indeed found to be also required for the RNAi response against RNA viruses in flies (Saleh et al., 2009). Other *in vitro* experiments also demonstrated that phagocytosis of bacteria that express dsRNA can induce RNAi (Rocha et al., 2011). Systemic RNAi in insects therefore does not necessarily require naked dsRNA but could also be triggered via phagocytosis of dsRNA-containing microorganisms, apoptotic cell fragments and exosomes (Rocha et al., 2011).

A few studies have followed the uptake of fluorescently labeled dsRNA by insect cells. Although dsRNA could be taken up by lepidopteran cell lines such as Sl2 (from *Spodoptera littoralis*) and Hi5 (from *Trichoplusia ni*), it could not trigger RNAi, indicating a barrier in the presentation of dsRNA to the RNAi machinery and not in uptake *per se* (Swevers and Smagghe, 2012). In the coleopteran *Diabrotica virgifera*, uptake of long dsRNA (240 bp) was demonstrated but not of siRNA (Bolognesi et al., 2012). The requirement of a minimum length of dsRNA for efficient uptake of dsRNA agrees with a study in S2 cells where maximum efficiency of silencing was achieved at a length of 200 bp (Saleh et al., 2006).

### Degradation of dsRNA/small RNA

The prime example of a nuclease interfering with RNAi efficiency is ERI-1, which degrades the 3′-overhangs of siRNAs and is a negative regulator of RNAi in nervous tissue in *C. elegans* (Kennedy et al., 2004). However, despite its conservation in insects and mammals, no role in RNAi was found for the ERI-1 ortholog in *Drosophila* (Kupsco et al., 2006). In general, nucleases can have both positive and negative effects on RNAi efficacy, as they can stimulate the further degradation of targeted RNAs after the initial cleavage by the RISC complex or inhibit RNAi through non-specific degradation of the dsRNA trigger or the small RNA guide. In *Drosophila*, several nucleases or proteins with nuclease motif with positive and negative effects on RNAi efficiency were indeed identified as components of RISC complexes and in functional genomic screens; examples are C3PO (Liu et al., 2009), Tudor-SN (Caudy et al., 2003), Zucchini and Squash (Pane et al., 2007), and Nibbler (Han et al., 2011).

In other insects, the activity of dsRNA-degrading activity in the hemolymph has been considered as a contributing factor to explain differences in RNAi efficacy between the cockroach *Blattella germanica* and the tobacco hornworm *Manduca sexta*. DsRNA is much more stable in cockroach hemolymph (high systemic RNAi efficiency) than in *Manduca* hemolymph (low systemic RNAi efficiency) (Garbutt et al., 2013).

In *Bombyx mori* (Lepidoptera), a non-specific DNA/RNA nuclease with dsRNase activity was purified from the midgut content that apparently is involved in the digestion of nucleic acids in the food (Arimatsu et al., 2007). Subsequent studies, however, showed a more broad expression pattern in other larval tissues indicating additional physiological roles. When expressed in the lepidopteran Hi5 cells, the “dsRNase” was found to digest intracellularly dsRNA, to co-localize with Dicer-2, and to inhibit RNAi of a luciferase reporter (Liu et al., 2012).

It can also be noted that the midgut content of lepidopteran insects is highly alkaline (pH > 11; Dow, 1992) which de-stabilizes unprotected RNA. DsRNA is indeed rapidly degraded by *Bombyx* midgut content (Liu et al., 2013). Also orthopterans and hymenopterans possess alkaline midgut contents (Ortego, 2012). On the other hand, coleopteran and hemipteran insects have slightly acidic midgut contents (Ortego, 2012) which is a more favorable environment for dsRNA with respect to pH.

### Innate immune response

In mammals, it is well-established that dsRNA in somatic cells triggers the interferon response and activates interferon-stimulated genes to establish an antiviral state in the cells (Sadler and Williams, 2008). Thus, dsRNA can act in mammals as a “pathogen-associated molecular pattern” (PAMP) that is recognized by “pattern recognition receptors” (PRRs) to trigger physiological responses for combating viral infections. Examples of PRRs for dsRNA include cytoplasmic RNA helicases like Retinoic acid-inducible gene I (RIG-I) and Melanoma differentiation-associated gene-5 (Mda-5), protein kinase R (PKR), 2′–5′ oligoadenylate synthetase (OAS) and Toll-like receptors (TLRs) that can bind various forms of DNA and dsRNA (Sadler and Williams, 2008). It was observed that interferon responses are induced by dsRNAs with length >30 bp while smaller siRNAs can enter the RNAi pathway with minimal side-effects (Elbashir et al., 2001).

In insects, it is generally assumed that a non-specific interferon response like in mammals does not exist and that long dsRNA molecules can be used to induce gene silencing by RNAi in a specific manner. However, there are indications that non-specific dsRNAs (like dsGFP that does not target any insect gene) can induce physiological effects in insect tissues. Injection of 10 μg of dsGFP in pupae of the Chinese oak silk moth *Antheraea pernyi* (corresponding to a dose of 5 ng/μl) resulted in the induction of *Hemolin*, an immune response gene (Hirai et al., 2004). More recently, it was observed that injection of non-specific dsRNA in *Bombyx* and *Manduca* larvae resulted in the increased expression of genes of the RNAi machinery, such as *Dicer-2* and *Ago-2* (Garbutt and Reynolds, 2012; Liu et al., 2013). In the silkmoth, dsRNA also caused down-regulation of *BmToll9-1*, which encodes a TLR, and up-regulation of *dsRNase*, a non-specific nuclease with dsRNase activity and a possible involvement in the RNAi response (Liu et al., 2012, 2013). Because the transcriptional response of *BmToll9-1* to dsRNA resembles the response to lipopolysaccharide, a known PAMP from bacteria, it was speculated that dsRNA could act as a PAMP in *Bombyx* and that induction of RNAi machinery and *dsRNase* genes reflects a defense mechanism against RNA viruses (Liu et al., 2013). It was indeed reported that *dsRNase* expression is induced following infection of *Bombyx* larvae with *Bombyx mori* cytoplasmic polyhedrosis virus (BmCPV), which is characterized by a genome of linear dsRNA segments (Wu et al., 2009; Mori and Metcalf, 2010). Whether induction of RNAi machinery genes by administration of non-specific dsRNA is a conserved response in non-lepidopteran insects is not clear: no effects were observed in the pea aphid, *Acyrthosiphon pisum* (Hemiptera) (Christiaens, 2013) while *Dicer-2* expression was induced in the cockroach, *B. germanica* (Lozano et al., 2012).

In crustaceans, treatment with non-specific dsRNA can induce an antiviral state which will protect the animals from subsequent virus infection (Robalino et al., 2004, 2005). A similar protection to virus infection by pretreatment with dsRNA is also observed in an insect cell line derived from the sandfly *Lutzomyia longipalpis* (Pitaluga et al., 2008).

Finally, it was found that in *Drosophila*, viral infection triggers the expression of *Vago*, a secreted protein with antiviral activity (“cytokine-like”) and that this induction requires *Dicer-2* (Deddouche et al., 2008). The helicase domain of Dicer-2 belongs to the same DExD/H-box helicase family as do the RIG-I–like receptors in mammals, which function as PRRs for dsRNA. The DExD/H-box helicase family therefore could represent an evolutionary ancient set of sensors that detect viral nucleic acids and direct antiviral responses (Deddouche et al., 2008).

### Persistent virus infection

Acute viral infections are characterized by a high viral replication rate and production of a large number of progeny which, however, is limited in time because it is terminated either by the death of the host or by the mounting of a successful antiviral immune response. Persistent infection of viruses, on the other hand, may result from an acute infection that is not cleared: low levels of replication can continue over longer periods of time while the ability to be transmitted to other organisms or to the offspring at low levels is maintained (Goic and Saleh, 2012). Several categories of persistent infections can be distinguished; during latent infections, no viral progeny is produced by the host during certain periods while chronic infections are characterized by a steady production of viral progeny, that can vary from low-level to high-level. Such persistent infections can have a beneficial effect on the host and in some cases the virus can be adopted by the host (endogenous viruses) (Sorrell et al., 2009; Goic and Saleh, 2012).

**Figure 1:**
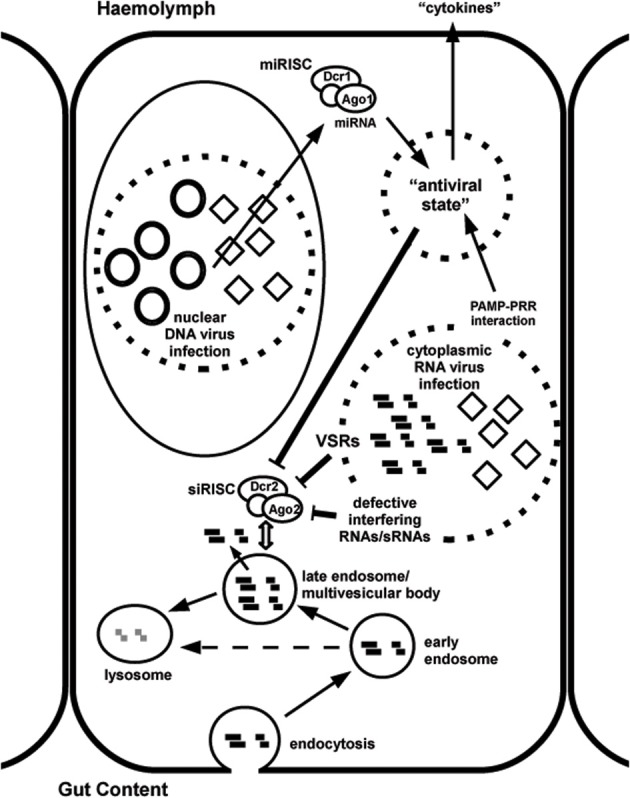
**Overview of uptake of dsRNA by the cell to trigger RNAi and possible mechanisms of interference by persistent RNA (or DNA) virus infection.** As an example, a gut epithelial cell is shown. Note that many of the proposed mechanisms are hypothetical. DsRNA is taken up from the gut content by endocytosis (“environmental RNAi”) and hypothetically may be presented, by an unknown mechanism, at the late endosomal stage to the RNAi machinery (siRISC complexes containing Dicer-2 and Ago-2). Alternatively, internalized dsRNA can be degraded in lysosomes. Cytoplasmic RNA virus infection can interfere with the RNAi machinery by the production of protein viral suppressors of RNAi (VSRs) and by the generation of large amounts of RNAs (including defective interfering RNAs) and small RNAs (sRNAs) that will “overflow” the RNAi machinery. Virus-derived pathogen-associated molecular patterns (PAMPs; for instance dsRNA) can also interact with pathogen recognition receptors (PRRs; for instance helicase domain of Dicer-2) to activate innate immune pathways that will establish a general “antiviral state” in the cells. If such an alternative pathway exists to control persistent virus infection, the maintenance costs for effective RNAi may become too high and deficiencies in the RNAi machinery may accumulate. The establishment of such hypothetical general “antiviral state” could also occur during nuclear DNA virus infection, for instance through the modulation of host miRNAs or the production of viral miRNAs (which are assembled in miRISC complexes containing Dicer-1 and Ago-1). The local antiviral response can hypothetically also result in the secretion of cytokine-like factors that will spread through the insect body to establish a systemic antiviral reaction.

The remainder of this review will focus on the question whether persistent virus infections can affect the efficiency of RNAi triggered by exogenous dsRNA in insects. This has indeed been observed in natural populations of *C. elegans*, where refractoriness to RNAi can be traced to persistent virus infection (Félix et al., 2011). Subsequently, we will look at the distribution of occurrence of viruses among different insect orders and see whether a correlation can exist between occurrence of viruses and systemic/environmental RNAi efficiency and will consider the mechanisms by which viruses could affect the RNAi process. Experimental systems will be proposed to test the hypothesis of inhibition of RNAi by persistent virus infection and its possible impact on the design of RNAi-based methods for control of insect pests will be discussed. An overview of the mechanisms by which persistent virus infection can affect the function of the RNAi machinery is presented in Figure 1.

## RNAi efficiency in natural isolates of *C. elegans*

Two natural isolates of the roundworms *C. elegans* and *C. briggsiae* were identified with unusual morphology in the intestinal cells that was caused by infection with two new species of nodavirus [(+)-ssRNA virus] (Félix et al., 2011). Interestingly, the natural isolate of *C. elegans* was resistant to RNAi of a somatically expressed gene while competent for dsRNA-mediated silencing in the germline. Because RNAi is an antiviral response in *C. elegans* (Lu et al., 2005; Schott et al., 2005; Wilkins et al., 2005), it was hypothesized that the defect in the somatic RNAi pathway was instrumental in the capability of the virus to accumulate to high levels and cause morphological alterations in the intestinal cells.

It is also observed that in the genus *Caenorhabditis* there exists a wide variability in susceptibility to systemic and environmental RNAi (Nuez and Félix, 2012). The pattern of sensitivity/insensitivity does not follow clearly the phylogenetic relationships. Different wild isolates of *C. elegans* showed also variation with respect to somatic and germline RNAi (Tijsterman et al., 2002; Félix, 2008). A driver for this variability may be the occurrence of dsRNAs from transposons (for intracellular RNAi) and pathogens such as viruses in the external environment and the intestinal lumen (for environmental RNAi) (as discussed by Félix, 2008; Nuez and Félix, 2012). Transposons or pathogens may lead to different selection pressures in even closely related species and to the observed rapid evolution of the RNAi machinery, particularly in the germ line for transposons and in the intestine for viruses (Nuez and Félix, 2012).

Outside the genus of *Caenorhabditis*, other nematode species were also found to be incapable of mounting an RNAi response (Pires da Silva and Sommer, 2004), although notable exceptions exist that even exhibit sensitivity to feeding of dsRNA (Shannon et al., 2008). The RNAi response can therefore be considered evolutionary very labile in nematodes since it forms part of the innate immune response and is characterized by a rapid evolutionary rate (Dalzell et al., 2011), a situation which may not be very dissimilar to the one encountered in insects.

## Virus infection in insects of different taxonomic groups

If persistent virus infection could affect RNAi efficiency, it is of interest to see which insects are most often associated with virus infection. In this analysis, virus-insect associations were searched in the database of insect pathogens, namely EDWIP (Ecological Database of the World's Insect Pathogens; Braxton et al., 2003), for the seven insect orders for which a good number of RNAi experiments have been reported (Lepidoptera, Diptera, Coleoptera, Hymenoptera, Orthoptera, Hemiptera and Dictyoptera) (Table 1).

**Table 1:** **Virus associations with insects of different taxonomic groups**.

**Insect order #species**		**Lepidoptera**	**Diptera**	**Coleoptera**	**Hymenoptera**	**Orthoptera**	**Hemiptera**	**Dictyoptera**
		**120,000**	**150,000**	**400,000**	**125,000**	**20,000**	**80,000**	**6000**
DNA virus	Baculoviridae	833	27	18	32	3	1	0
	Iridoviridae	87	58	19	3	3	5	0
	Parvoviridae	66	6	6	0	2	0	0
	Poxviridae	27	9	20	4	8	0	0
	Other	14	0	1	2	0	0	0
	Total (%)	1027 (82%)	100 (8%)	64 (5%)	41 (3%)	16 (1%)	6 (0.5%)	0 (0%)
RNA virus	Reoviridae	218	42	5	8	0	4	0
	Picornaviridae (^*^)	12	1	0	3	2	5	0
	Nodaviridae	7	6	9	1	0	0	0
	Tetraviridae	19	0	0	0	0	0	0
	Other	0	1	0	11	1	1	0
	Total (%)	256 (72%)	50 (14%)	14 (4%)	23 (6%)	3 (1%)	10 (3%)	0 (0%)
Total (%)		1283 (80%)	150 (9%)	78 (5%)	64 (4%)	19 (1%)	16 (1%)	0 (0%)

*Indicated is the number of reported cases in the EDWIP database. Note that data in the database have not been updated since 2001 and therefore may not exactly reflect the current status of virus occurrences*.

* *Although mentioned in the database as Picornaviridae, it is noted that now officially members of Picornaviridae are composed from vertebrate viruses. For insects, “Picornaviridae” corresponds to Iflaviridae and Dicistroviridae*.

While this analysis may give a reasonable indication of the occurrence of virus infections in insects of different taxonomic groups, it also has some important limitations. The database reports how many different viruses have been identified in different insect species, but does not take into account how widespread/prevalent these viruses are amongst individuals of specific insect species. Moreover, there is likely to be a major sampling bias, in that insects that are of economic or medical importance are likely to be sampled much more frequently than other insects. Thus, more extensive sampling is needed to strengthen the results presented in this preliminary analysis. Nevertheless, a clear correlation can be found between numbers of recorded virus infections and perceived systemic/environmental RNAi efficiency in species of particular taxonomic groups.

The vast majority of viruses were found to be associated with Lepidoptera (Table 1). While this mostly reflects baculovirus infections (~50%), it is noted that 72% of the RNA virus infections also occurs in lepidopteran insects. Much less viral infections (~10%) are reported in Diptera, and most of these occur in mosquitoes. In the database, no arthropod-borne viruses (arboviruses; that transmit viral disease to humans and other mammals; see also further below) are present and the number of virus infections in mosquitoes therefore should be estimated as higher.

Equally striking is the low occurrence of virus infections in coleopterans, despite their high abundance of species (Table 1). Other orders with efficient RNAi, such as Hymenoptera, Orthoptera and Dictyoptera, are also characterized by low virus occurrences. In the case of cockroaches (Dictyoptera), no virus infections were reported in the database. On the other hand, a report characterizing a densovirus infection in *B. germanica* has recently been published (Mukha et al., 2006).

Also a low number of virus occurrences were found in the database for Hemiptera (Table 1). As plant-sucking insects, however, hemipterans are obligatory associated with prokaryotic symbionts that provide them with essential amino-acids (Douglas, 2003; Baumann, 2005). It has been observed that symbiont associations can affect virus transmission (Moreira et al., 2009; Mousson et al., 2012). Hemipterans, mainly aphids but also whiteflies, are also known to transmit viral diseases among plants (insect-borne plant viruses; Sylvester, 1989; Brown and Czosnek, 2002; Chougule and Bonning, 2012).

Regarding RNA viruses, the highest number found in insects corresponds to the Reoviridae, which include the cytoplasmic polyhedrosis viruses (CPVs; genus *Cypovirus*). Reoviridae are characterized by a segmented genome of 10–12 linear dsRNA fragments (Mori and Metcalf, 2010). In *Bombyx mori*, BmCPV is a major pathogen and causes enormous damage to sericulture (Wu et al., 2009). A characteristic of CPV, like nucleopolyhedrosis virus (NPV) or baculovirus, is the production of polyhedrin protein which crystallizes in the cells to form occlusion bodies and allows viral transmission among larvae in the environment (Payne and Mertens, 1983). After ingestion, the polyhedra dissolve in the alkaline environment of the gut to release viral particles. Usually the infection is limited to the gut epithelium although spread to other tissues has been observed. CPV infection in lepidopteran larvae is a slow process and often allows infected larvae to undergo spinning and pupation; because of the low virulence, persistent infections can be easily established (Pirofski and Casadevall, 2012). However, little is known how these persistent infections can influence the physiology of the larvae and whether they could affect the RNAi pathway.

## How can virus infection interfere with RNAi efficiency?

Because in insects the RNAi machinery is harnessed to combat viral infection, viruses have evolved strategies to interfere with the function of the RNAi machinery. An antiviral defense mechanism for RNAi was first demonstrated in plants (Ratcliff et al., 1997) and in *C. elegans* (Lu et al., 2005; Schott et al., 2005; Wilkins et al., 2005). In *Drosophila*, the siRNA pathway has been identified as a major antiviral defense mechanism since mutants in the core siRNA components Dicer-2, Ago-2, and R2D2 are more susceptible to viral infection, as demonstrated by the accumulation of high viral titers and the premature death of the infected flies (van Rij et al., 2006; Wang et al., 2006; Vodovar and Saleh, 2012). The role of RNAi in antiviral defense has also been firmly established in mosquitoes (Keene et al., 2004; Campbell et al., 2008; Sanchez-Vargas et al., 2009). The involvement of the RNAi pathway is also confirmed by the identification of virus-derived siRNAs (viRNAs) that were shown to play a key role in controlling the pathogenic potential of virus infection (Myles et al., 2008; Scott et al., 2010).

Regarding the involvement of the siRNA pathway, it was also shown that Ago-2, Dcr-2, and R2D2 belong to the fastest evolving genes, similar to immunity genes, and in sharp contrast to the high conservation of miRNA pathway genes (Obbard et al., 2006). Moreover, viral suppressors of RNAi (VSRs) target the function of the siRNA, but not the miRNA pathway (Li et al., 2002; van Rij et al., 2006; Wang et al., 2006; Berry et al., 2009). It appears that the siRNA-based RNAi pathway is involved in an evolutionary arms race against attacking and constantly evolving viral invaders (Ding and Voinnet, 2007).

Recent studies have also established that the piRNA pathway can function as an antiviral defense mechanism (Zambon et al., 2006; Brackney et al., 2010; Vodovar et al., 2012). In the case of *Aedes albopictus*-derived C6/36 cells, which do not contain functional Dicer-2, piRNAs were proposed to silence viral infection (Scott et al., 2010). Finally, it has recently been established that baculoviruses can produce miRNAs and that virus infection can affect host miRNA expression (Singh et al., 2012; Wu et al., 2013). Thus, miRNAs could also be involved in the evasion of the innate immune response (to the benefit of the virus) and the establishment of an antiviral state in the cells (to the benefit of the host) (see also further below).

Several mechanisms can be predicted by which virus infection can interfere with RNAi. These include the expression of viral suppressors of RNAi (VSRs), the accumulation of large amounts of viral RNAs and small RNAs that will overflow the RNAi machinery, the modulation of expression of host miRNAs and expression of viral miRNAs and the induction of a general antiviral state in the infected insects (Figure 1).

### Viral suppressors of RNAi (VSRs)

VSRs for which the mechanism of inhibition of RNAi is rather well-established include the B2 protein from Flock house virus (FHV; Nodaviridae) and the 1A proteins from *Drosophila* C virus (DCV; Dicistroviridae) and cricket paralysis virus (CrPV; Dicistroviridae) (Li et al., 2002; Wang et al., 2006; Aliyari et al., 2008; Nayak et al., 2010). B2 can bind tightly to dsRNA and siRNA and therefore inhibit RNAi by sequestration of dsRNA trigger or siRNA effector (Lingel et al., 2005). It also can form a complex with the viral RdRp and therefore associate with dsRNA replication intermediates immediately upon their emergence (Aliyari et al., 2008). FHV B2 also interacts with the PAZ domain of Dicer which is thought to contribute to the suppression of siRNA biogenesis (Singh et al., 2009).

Also the 1A protein of DCV could bind efficiently to dsRNA and, to a lesser extent, siRNA (van Rij et al., 2006). On the other hand, 1A protein of CrPV did not bind dsRNA/siRNA but could block the catalytic activity of RISC (target cleavage) by direct interaction with Ago-2 in *Drosophila*-derived S2 cells (Nayak et al., 2010). The virulence of both viruses could be correlated with the capacity to inhibit RNAi in S2 cells: while CrPV-1A, unlike DCV-1A, effectively blocked silencing induced by transfection of both long dsRNA as well as siRNA, it is also observed that DCV can establish persistent infection in contrast to CrPV which is highly lytic (Nayak et al., 2010). Such data fit with observations that arboviruses that are vectored by mosquitoes are thought not to contain powerful VSRs (Myles et al., 2008). To increase transmission rates, it is considered more beneficial to the arbovirus to minimize effects on mosquito survival than to maximize infection rate at the cost of decreased survival. Recombinant arboviruses that are engineered to express exogenous VSRs indeed cause lethal mosquito infections (Cirimotich et al., 2009). Examples of such arboviruses that cause asymptomatic infections in hematophagic insects, but are responsible for severe incapacitating diseases in mammals include Rift Valley fever virus, Chikungunya virus, Dengue virus, yellow fever virus (vectored by *Aedes aegypti*), O'nyong'nyong virus (vectored by *Anopheles spp*), Japanese encephalitis virus, West Nile virus (vectored by *Culex spp*), vesicular stomatitis virus (vectored by the sand fly *Phlebotomus spp*) and Bluetongue virus (vectored by biting midges in the genus *Culicoides*). More recently, however, a few publications appeared that, contrary to expectations discussed above, indicate that flaviviruses such as Dengue and West Nile virus can encode RNAi suppressors, such as the non-coding sfRNA and the non-structural protein NS4B (Schnettler et al., 2012; Kakumani et al., 2013). Further studies are needed to determine to what extent RNAi suppression is required for transmission of flaviviruses and other arboviruses in mosquitoes and how it compares with the control of viruses with higher virulence.

Recently, the VP1 protein of Noravirus, a picorna-like virus, was identified as an RNAi suppressor (van Mierlo et al., 2012). When added to *Drosophila* embryonic extracts, VP1 was able to inhibit cleavage of RNA targets, indicating that VP1 inhibits the effector phase of RNAi (RISC complexes). Another study, on the other hand, showed that Nora virus infection was not affected by *Dicer-2, Ago-2 r2d2* and *piwi* mutations in flies (Habayeb et al., 2009). While Noravirus can establish persistent infections in *Drosophila* that are virtually asymptomatic, it was also observed that populations of flies can show high variability in viral titers (Habayeb et al., 2009).

Interestingly, an ascovirus that infects *Heliothis virescens* (HvAV; DNA virus) encodes an RNase III enzyme that is capable of degrading dsRNA and siRNA and is essential for infection (Hussain et al., 2010). Furthermore, HvAV RNase III can inhibit RNAi-mediated silencing of a reporter gene, suggesting that it may be involved in the suppression of the RNAi response during infection (Hussain et al., 2010).

### Decoy mechanisms: production of large amounts of viral RNAs and small RNAs

In *Drosophila* S2 cells, FHV can establish persistent infections without causing pathogenic effects (Flynt et al., 2009; Goic et al., 2013). The virions that are produced in the persistently infected cells, however, are still capable to mount robust infection in uninfected cells and encode an intact B2 inhibitor protein. Interestingly, the S2 cell lines that are persistently infected, produce considerable amounts of RNA2 (genomic RNA encoding the capsid protein) and RNA3 (a subgenomic RNA that produces the B2 suppressor) but very few RNA1 (genomic RNA encoding RdRp). While depletion of Dicer-2 and Ago-2 results in an increase in viral replication in the persistently infected cells, deep sequencing also identified a significant number of FHV-derived small RNAs (Flynt et al., 2009). The majority of vsiRNAs, however, map to discrete regions (“hotspots”) of the virus. Functional tests established that the abundant viral siRNAs produced during latent viral infection are largely ineffective at silencing complementary transcripts. Because these vsiRNAs were not found to be methylated, it was proposed that they were not associated with Ago-2-containing RISC complexes (Flynt et al., 2009). Thus, Dicer-2-mediated “dicing” of double-stranded replication intermediates may have a direct role in controlling persistent FHV infection, in the absence of RISC activation by the produced siRNAs.

In plants, the generation of defective interfering RNAs can affect the accumulation of virus and viral symptoms in the infected plants (Pathak and Nagy, 2009). They consist of RNA sequences that are derived from the virus but which need proteins of the virus for their replication. Defective interfering RNAs are referred to as “interfering” because they attenuate the viral symptoms which in turn will favor persistent infections. Defective interfering RNAs can result in the accumulation of large amounts of siRNAs which could “overflow” the capacity of the VSRs produced by the virus and trigger a potent RNAi response (Havelda et al., 2005). Also, in mammals, viruses can employ a “decoy” mechanism to inhibit RNAi; for instance, the VA1 non-coding RNA from adenovirus that is expressed at very high levels can inhibit Dicer function (Lu and Cullen, 2004). It would be interesting to investigate whether insect viruses can interfere with the RNAi machinery through similar mechanisms.

### Establishment of a general antiviral state in persistently infected cells

Establishment of a persistent virus infection can result in changes in the physiology of the infected cells that are independent of the RNAi mechanism. This is the case when the infected cells control the viral infection by another (so far hypothetical) antiviral response pathway. The prevalence of the other antiviral pathway(s) may become very effective in controlling infection and subsequently render the RNAi pathway obsolete. In that case, maintenance of effective RNAi machinery against invading viruses could be selected against and mutations would start to accumulate in the RNAi machinery. Below follows a discussion of alternative mechanisms by which insects may control viral infections and that could lead to a selection against a functional RNAi machinery.

#### Classical innate immunity pathways

While a clear role has been established for RNAi to control viral infections, other studies suggest that the other innate immune pathways, Toll, Imd and Jak-STAT, that play major roles during bacterial and fungal infections, are not involved to a great extent to control RNA virus infections in *Drosophila* and mosquitoes (Merkling and van Rij, 2013). While effects of virus infection on the expression of the different innate immune pathways were observed and knockdown of components in each pathway can result in higher viral loads (for examples see Dostert et al., 2005; Xi et al., 2008; Costa et al., 2009; Souza-Neto et al., 2009), no clear picture emerges and it can be asked whether the variability is caused by different experimental approaches, some unique properties of different viruses or the different host species under investigation (Merkling and van Rij, 2013).

#### Evidence for unique responses against viral infection

In *Drosophila*, infection with DCV triggers expression of approximately 150 genes (Deddouche et al., 2008). Since some genes, such as *vir-1*, are known targets of Jak-STAT signaling, a pathway activated by cytokines in mammals, it was proposed that DCV infection could also trigger a cytokine-like molecule in *Drosophila* that would induce Jak-STAT signaling as a secondary response (Deddouche et al., 2008). One candidate cytokine-like molecule was identified as Vago, a 160 AA secreted protein with characteristic Cys-rich motif, and its production during DCV infection was shown to depend on Dicer-2 (but not Ago-2 or R2D2 or the Imd and Toll pathways). However, whether Vago can induce Jak-STAT signaling as a secondary systemic response in other cells of the organism still remains to be established. Because the helicase domain of Dicer-2 is phylogenetically related to the RIG-1 and Mda5, which function as PRRs for dsRNA in mammals (Sadler and Williams, 2008), it was proposed that Dicer-2, through its helicase domain, can function as a PRR in *Drosophila*, independently from its dicer activity in the RNAi pathway (Deddouche et al., 2008). Vago is not conserved in other insect groups, but it will be interesting to see whether molecules that can act as cytokines are induced upon virus infection in other insects.

Microarray analysis and suppression subtractive hybridization techniques were also used to identify genes that become differentially expressed in the midgut of *Bombyx mori* upon BmCPV (Reoviridae) infection (Wu et al., 2009, 2011). The transcriptional response, however, did not reveal an involvement of the classical innate immunity genes (Toll, Imd and Jak-STAT pathways). One of the up-regulated genes was found to encode a secreted factor with homology to insulin-like growth factor-binding proteins (Gao et al., 2012c). Other interesting factors that may play a role in BmCPV infection include LIM and SH3 containing protein 1 (LASP1), ganglioside-induced differentiation-associated protein 1 (GDAP1), non-specific alkaline nuclease (which can also be induced following dsRNA injection, see above), Inhibitor of apoptosis protein (IAP; which decreases upon infection and therefore leads to apoptosis; Narayanan, 2004), serpin5 (a putative negative regulator of the Toll pathway), lipase-1 (which is also active against NPV; Ponnuvel et al., 2003), heat-shock proteins and others (Wu et al., 2009, 2011; Gao et al., 2012a,b).

If persistent viral infections become controlled by other (unknown) mechanisms than RNAi, it is possible that the need for the maintenance of an active RNAi response against exogenous dsRNA disappears and that mutations accumulate in the RNAi machinery. For instance, frame-shift mutations in *r2d2* of *Bombyx mori* have been observed (Swevers et al., 2011), while *Bombyx* larvae are often persistently infected with BmCPV (Wu et al., 2009). Viral infections are also correlated with defective RNAi in *C. elegans* (Félix et al., 2011). Thus, the interaction between host immune response and persistent viral infection could result in a permanent antiviral state with consequences for the functioning of the RNAi machinery. Deficiencies in RNAi are not necessarily restricted to the core RNAi machinery but could affect systemic/environmental RNAi processes as well as uptake of dsRNA and the abundance of dsRNA-degrading enzymes.

#### Nucleic acid-based acquired immunity and its potential interaction with the core RNAi machinery

In another study of persistent infection of S2 cells by FHV, it was found that during establishment of viral persistence, RNA viruses were reverse-transcribed into cDNA by host reverse transcriptases (Goic et al., 2013). Sequencing established that the viral DNA forms, which were heavily reorganized through non-homologous recombination, were fused to long terminal repeat (LTR) retrotransposons; those FHV-retrotransposon DNA chimera's were transcribed and processed to small RNAs to control viral infection (Goic et al., 2013). It was not determined whether the viral DNA forms exist as extrachromosomal entities or become integrated in the cellular chromosomes; however, it can be noted that an extra-viral source of vsiRNAs is created which does not only control the current infection, but possibly also other infections in the future once the current one would be cleared. It is also possible that such cellular loci that produce vsiRNAs would exert additional pressure on the RNAi machinery and make the latter less sensitive to other sources of dsRNA.

#### Manipulation of host gene expression by virus infection possibly resulting in diminished RNAi efficiency

It can be hypothesized that persistently infecting viruses also could manipulate gene expression in host cells that would result in a diminished RNAi response. This could be the case with DNA viruses such as baculoviruses and iridoviruses. Reduction in RNAi efficiency is also relevant in the case of DNA virus infection since it is documented that not only RNA viruses but also DNA viruses can trigger the RNAi response in insects. For instance, *Drosophila Dcr2* and *Ago-2* mutants are more susceptible to the invertebrate iridescent virus 6 (Iridoviridae) (Bronkhorst et al., 2012). In such case, vsiRNAs originate from hotspots in the genome where overlapping sense and antisense transcripts are produced. Similar observations were made during infection of a baculovirus, *Helicoverpa armigera* single nucleopolyhedrovirus (HaSNPV) (Jayachandran et al., 2012). Small RNA reads could be matched to hotspots in the baculoviral genome, corresponding to late genes that are involved in replication and dispersal of the virus. Knockdown of Dicer-2 resulted in a decrease in late gene expression, while knockdown of both Dicer-1 and Dicer-2 caused a modest, but significant, increase in viral DNA replication (Jayachandran et al., 2012).

Nuclear DNA viruses, such as baculoviruses, on the other hand, are also known to produce miRNAs (Singh et al., 2010, 2012). Cytoplasmic viruses are not expected to encode miRNAs since processing of the primary miRNA transcript is required by the nuclear Drosha/Pasha complex. Viruses with relatively small RNA genomes are also not likely to encode a miRNA because miRNA processing would result in the complete destruction of the primary miRNA transcript with the exception of the mature miRNA. Viral miRNAs act by suppression of both viral and cellular mRNAs to interfere with apoptosis and to evade the host immune response in order to prolong the life of the infected cells and to maximize the viral replication potential (Umbach and Cullen, 2009). Of interest is that miRNAs are also involved in the regulation of the latency phase of virus infection in the case of herpesviruses (Umbach et al., 2008; Tang et al., 2009). MiRNAs could therefore play an important role in the establishment of persistent viral infections.

*Bombyx mori* nuclear polyhedrosis virus (BmNPV; Baculoviridae) has been shown to produce several miRNAs (Singh et al., 2010), of which one, bmnpv-miR-1, can inhibit the expression of the GTP-binding protein Ran, a co-factor of the Exportin-5-mediated nucleocytoplasmic transport machinery (Singh et al., 2012). Blocking Ran expression results in decreased nucleocytoplasmic transport of miRNA precursors and, consequently, in repression of biogenesis of host miRNAs, including those that target BmNPV mRNAs. More specifically, the cellular miRNA bmo-miR-8 has multiple binding sites on the mRNAs of *ie-1* and other important BmNPV genes and its decrease therefore results in the increase of virus accumulation (Singh et al., 2012). It was also observed that silencing of Dicer-2 (but unexpectedly not Dicer-1) could lead to an increase in viral load in the infected larvae (Singh et al., 2012).

In case of persistent infection of baculovirus, it can therefore be expected that the miRNA profile in infected cells shows dramatic changes. Given the capacity of miRNAs to regulate many mRNA targets (Lim et al., 2005; Selbach et al., 2008), a significant change in the physiology of the cells is expected. It would be interesting to investigate if such infections also would lead to diminished expression or function of core RNAi factors, dsRNA uptake components or nucleases.

## A testable hypothesis

To test whether persistent virus infection results in changes of RNAi efficiency, two complementary approaches can be used. In the first approach, persistent viral infections will need to be established in insects that have high RNAi efficiency and are virus-free. Examples of such insects are locusts, cockroaches and beetles, in which high RNAi efficiency was reported (Mané-Padrós et al., 2010; Zhu et al., 2011; Wynant et al., 2012). These experimental animals need subsequently to be checked for virus infection by deep sequencing techniques (Wu et al., 2010; Liu et al., 2011; Ng et al., 2011). A suitable virus to establish viral infections may be FHV (Nodaviridae), which has a broad host range and capable of infection of lepidopteran, dipteran, hemipteran and coleopteran insects (Dasgupta et al., 2003, 2007). It would be interesting to see whether FHV or a related nodavirus can infect locusts or cockroaches. An alternative virus for orthopteran insects could be the CrPV, which was isolated from Australian field crickets (Orthoptera: Gryllidae) (EDWIP database; Braxton et al., 2003). Another interesting experimental system would be the acquisition of arbovirus infection in hematophagous mosquitoes. As discussed earlier, arbovirus infection in mosquitoes resembles persistent infection, since they are largely asymptomatic (Myles et al., 2008; Cirimotich et al., 2009). It remains to be tested whether the silencing efficiency of mosquito genes through RNAi alters when arbovirus infections become more established.

A second approach would consist of a population of persistently infected insects that becomes subsequently cleared from virus. For instance, it is known that many populations of *B. mori* are persistently infected with the dsRNA virus BmCPV (Wu et al., 2009). Because transgenesis techniques have been developed for *Bombyx* (Tamura et al., 2000; Tomita et al., 2003), strains could be generated that express RNA hairpin genes that target BmCPV, a technique that has been used to combat baculovirus infection (Kanginakudru et al., 2007). Alternatively, different silkmoth strains could be screened for their propensity to be infected by BmCPV or individual larvae tested for presence of BmCPV infection and isolated from the rest of the population. Larvae that became virus-free can then be compared with persistently infected larvae with respect to gene silencing through RNAi. In addition, deep sequencing needs to be applied to confirm any additional virus infections that might occur simultaneously. Finally, if clear differences are observed, an extended analysis can be performed to identify the mechanism of RNAi sensitivity/refractoriness.

## Impact on strategies of insect pest control based on RNAi

For coleopteran pests, such as the corn rootworm, *D. virgifera*, and the Colorado potato beetle, *L. decemlineata*, RNAi-based control methods offer a real alternative to other control methods (Gordon and Waterhouse, 2007; Price and Gatehouse, 2008; Huvenne and Smagghe, 2010; Swevers and Smagghe, 2012). Because the strategy of RNAi targeting essential genes of insect pests has a mode of action which is independent from that of Bt toxins, it can be used for the creation of “pyramided” transgenic crops, where the action of Bt toxin is complemented by RNAi (Moar et al., 2010).

As for every method for insect control, however, the rise of insecticide resistance is always a major issue. It has been argued that resistance against dsRNA or RNA hairpins might be difficult to occur because long dsRNAs can still function effectively, even when multiple mutations have accumulated in the target sequence (Swevers and Smagghe, 2012). However, the RNAi machinery that is dedicated to defense against exogenous dsRNA is considered to be dispensable (Shabalina and Koonin, 2008) and could therefore mutate quickly if maintenance costs are too high and alternative mechanisms evolve against RNA virus infections. Persistent virus infection may appear as a major mechanism that inactivates RNAi (a correlation between persistent virus infection and RNAi deficiency in the soma is observed in natural isolates of *C. elegans*; Félix, 2008; Félix et al., 2011; Nuez and Félix, 2012). Thus, the rise of resistance in insect pest populations that are managed by RNAi-based techniques will give the opportunity to evaluate the importance of the acquisition of persistent viral infections as a resistance mechanism.

### Conflict of interest statement

The authors declare that the research was conducted in the absence of any commercial or financial relationships that could be construed as a potential conflict of interest.
